# Is age an independent risk factor for perioperative mortality and morbidity after radical prostatectomy? Analysis of the American college of surgeons national surgical quality improvement program database

**DOI:** 10.1080/2090598X.2020.1721165

**Published:** 2020-02-11

**Authors:** Ali Merhe, Mohammad Hout, Nassib Abou Heidar, Jose M. El-Asmar, Rola Jaafar, Aurelie Mailhac, Hani Tamim, Rami Nasr

**Affiliations:** a Department of Surgery, Division of Urology, American University of Beirut Medical Center, Beirut, Lebanon; b Biostatistics and Clinical Research Unit, Faculty of Medicine, American University of Beirut Medical Center, Beirut, Lebanon

**Keywords:** Age, morbidity, mortality, prostatectomy, prostate cancer

## Abstract

**Objectives:**

To assess the safety and surgical outcomes of radical prostatectomy (RP) when looking at age as an independent risk factor of perioperative mortality and morbidity.

**Patients and methods:**

A retrospective cohort study was performed using American College of Surgeons National Surgical Quality Improvement Program database. Patients who underwent a RP from 2008 to 2015 were identified. They were divided into three groups based on their age 15 group at the time of surgery. Patients’ characteristics were compared across the three following age groups: 74 years. The correlation between the three different age groups and their respective 30-day postoperative mortality and morbidity were assessed using logistic regression. Unadjusted and adjusted odds ratios (ORs) were estimated.

**Results:**

A total of 43025 patients were identified, 81.7% were aged 74 years. Overall, 102 patients died in the 30-day postoperative period. Univariate and multivariate analysis showed a significant increase in the 30-day postoperative mortality from 0.1% to 0.4% to 1.3% in the three different age groups 74 years, respectively. In addition, there was a significant increase in postoperative complications in the group of patients aged >74 years. A higher risk of complications 25 related to cardiac (OR 2.18 in age group 70–74 vs OR 7.45 in age group >74 years), respiratory (OR 2.36 vs OR 5.91), neurological (OR 2.28 vs OR 3.44), wound infections (OR 1.49 vs OR 3.25), and sepsis (OR 1.54 vs OR 2.64) were seen with the youngest group taken as a reference.

**Conclusion:**

Age is an independent risk factor for perioperative mortality and morbidity after RP in elderly patients. Therefore, age should be considered in the decision making of therapeutic options for patients with prostate cancer.

**Abbreviations:**

BMI: body mass index; CNS: central nervous system; SIOG: International Society of Geriatric Oncology; SEER: Surveillance, Epidemiology, and End Results; ACS: American College of Surgeons; NSQIP: National Surgical Quality Improvement Program; OR: odds ratio

## Introduction

Prostate cancer (PCa) is the most commonly diagnosed male cancer; it is one of the leading causes of cancer mortality worldwide [1,2]. Due to the increase in men’s life-expectancy, diagnosing PCa at an older age has increased significantly and is expected to continue to increase. There are several therapeutic regimens available for elderly men diagnosed with PCa ranging from active surveillance (AS) or watchful waiting, focal therapy, radiation therapy, and surgery. A surgical approach, radical prostatectomy (RP), entails the possibility of perioperative morbidity and mortality, especially in the elderly population.

Most studies exploring treatment regimens for PCa have focussed on men aged <75 years. However, in men with a life-expectancy >10 years, Richstone et al. [3] demonstrates the importance of careful patient selection for RP. Carefully selected men that are aged >70 years undergoing RP or radiotherapy have similar oncological outcomes and quality adjusted life-expectancy when compared to younger men. As such, patient selection as well as treatment modality selection is still uncertain and multifactorial [3].

There is a general tendency in PCa to undertreat the disease owing to the fact that it is generally an indolent disease; however, the International Society of Geriatric Oncology (SIOG) recommends that older patients with PCa should be managed according to their general health status and comorbidities rather than age alone [4–8]. Furthermore, an increased age of diagnosis is associated with more aggressive disease characteristics [9].

RP remains the ‘gold standard’ for the treatment of localised PCa, yet perioperative complications remain a major concern in regards to the surgical option in treating PCa, especially in elderly patients.

In our present analysis of the American College of Surgeons (ACS) National Surgical Quality Improvement Program database (NSQIP), we compared postoperative mortality and morbidity in patients undergoing RP for PCa in different age groups in order to determine whether age is an independent risk factor of perioperative mortality and morbidity.

## Patients and methods

**Figure 1.**
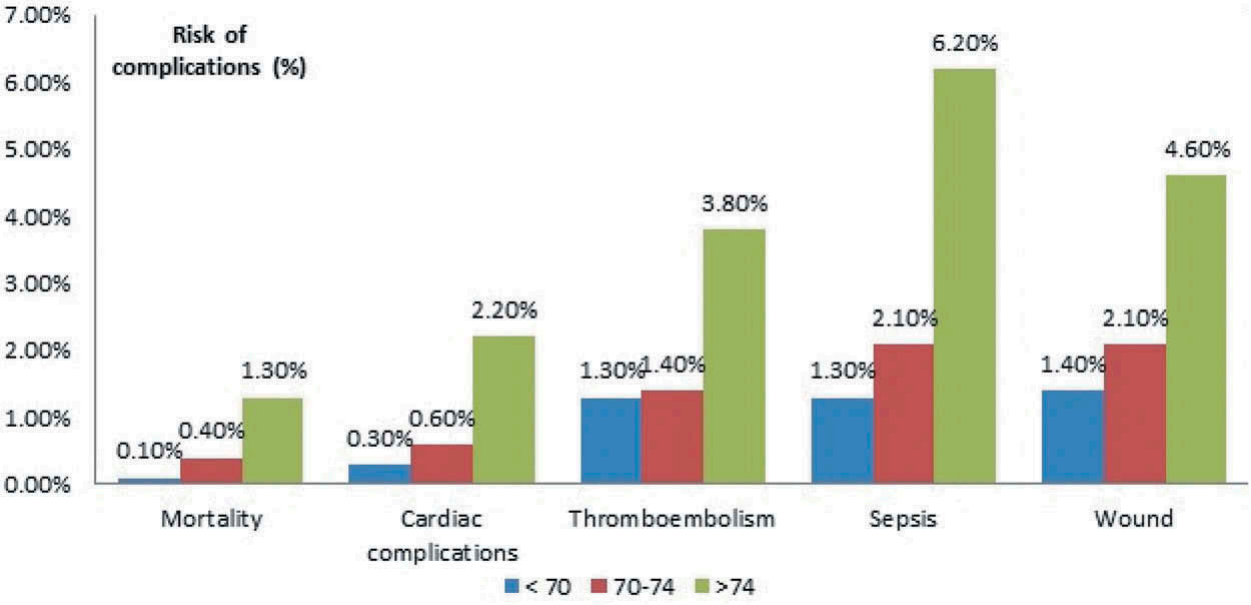
Risk of complications between different age groups.

Our retrospective cohort used data from the ACS NSQIP database (Figure 1). The database is a validated registry of outcomes on 30-day postoperative surgical mortality and morbidity from participating hospitals around the USA [10,11]. It includes data on demographics, perioperative variables, and 30-day postoperative outcomes for adult patients undergoing major surgery. A team of trained surgical clinical reviewers collected patient data. In addition, a comprehensive training for data review, regular conference calls, and annual meetings ensure proper quality of the data [12].

Patients who underwent a RP (open, laparoscopic, robot-assisted) from 2008 to 2015 were identified from the ACS NSQIP. Patients’ were divided into three groups based on their age at the time of RP. Patients’ characteristics were compared across the three following age groups: <70, 70–74, and >74 years. The correlation between the three different age groups and their respective 30-day postoperative mortality and morbidity was assessed using logistic regression. Frequency and percentage were used to describe categorical variables, whereas mean and standard deviation (SD) were used for continuous ones. Categorical variables were compared across the aforementioned age groups using the chi-squared test. ANOVA was used for the continuous ones. The lowest age category was set as a reference; separate logistic regression models were used for each outcome, upon which unadjusted and adjusted odds ratios (ORs) were estimated using the youngest age group as the reference group. Clinically potential confounders were considered for the multivariate analysis of each of the outcomes. All analyses were performed using Statistical Analysis System (SAS) (SAS Institute Inc., Cary, NC, USA). *P* values were two-sided and statistical significance was set at *P* < 0.05.

## Results

Over a span of 7 years, from 2008 to 2015, 43 025 patients underwent a RP in the USA. This included open, laparoscopic and robot-assisted surgery. In all, 81.7% of patients were aged <70 years, 12.6% were aged 70–74 years, and 5.5% were aged >74 years. Comorbidities amongst patients at 30-days preoperatively were evaluated. They ranged from no comorbidities to hypertension (51.6%), obesity (body mass index [BMI] >30 kg/m^2^, 35.4%), diabetes (12.7%), chronic obstructive pulmonary disease (2.5%), and congestive heart failure (0.1%) (Table 1).

**Table 1.** Demographics.

Variable	All (*N* = 43,025)	Age group, years			*P*
		<70 (*n* = 35,187)	70–74 (*n* = 5451)	>74 (*n* = 2387)	
*n/N* (%)					
Race					
White	31,747/37,036 (85.7)	25,733/30,416 (84.6)	4095/4551 (90.0)	1919/2069 (92.8)	<0.001
African-American	4229/37,036 (11.4)	3854/30,416 (12.7)	293/4551 (6.4)	82/2069 (4.0)	
Others	1060/37,036 (2.9)	829/30,416 (2.7)	163/4551 (3.6)	68/2069 (3.3)	
ASA classification					
I	1577/42,953 (3.7)	1432/35,125 (4.1)	121/5445 (2.2)	24/2383 (1.0)	<0.001
II	25,012/42,953 (58.2)	21,470/35,125 (61.1)	2709/5445 (49.8)	833/2383 (35)	
III	15,832/42,953 (36.9)	11,891/35,125 (33.9)	2526/5445 (46.4)	1415/2383 (59.4)	
IV	532/42,953 (1.2)	332/35,125 (1.0)	89/5445 (1.6)	111/2383 (4.7)	
Transfusion >4 units PRBCs in 72 h before surgery	99/43,025 (0.2)	47/35,187 (0.1)	16/5451 (0.3)	36/2387 (1.5)	<0.001
Functional health status prior to current illness					
Independent	42,750/42,905 (99.6)	34,986/35,086 (99.7)	5409/5434 (99.5)	2355/2385 (98.7)	<0.001
Partially dependent	135/42,905 (0.3)	86/35,086 (0.3)	22/5434 (0.4)	27/2385 (1.1)	
Totally dependent	20/42,905 (0.0)	14/35,086 (0.0)	3/5434 (0.1)	3/2385 (0.1)	
Hypertension requiring medication	22,213/43,025 (51.6)	17,329/35,187 (49.3)	3331/5451 (61.1)	1553/2387 (65.1)	<0.001
Current smoker within 1 year	5857/43,025 (13.6)	5203/35,187 (14.8)	485/5451 (8.9)	169/2387 (7.1)	<0.001
BMI ≥30 kg/m^2^	15,157/43,025 (35.4)	12,913/35,187 (36.9)	1668/5451 (30.7)	576/2387 (24.2)	<0.001
Diabetes mellitus with oral agents or insulin	5445/43,025 (12.7)	4146/35,187 (11.8)	881/5451 (16.2)	418/2387 (17.5)	<0.001
Systemic sepsis	121/43,025 (0.3)	83/35,187 (0.2)	16/5451 (0.3)	22/2387 (0.9)	<0.001
Days from hospital admission to operation					
0	42,319/43,025 (98.4)	34,746/35,187 (98.8)	5340/5451 (98.0)	2233/2387 (93.6)	<0.001
1	479/43,025 (1.1)	313/35,187 (0.9)	69/5451 (1.3)	97/2387 (4.1)	
>1	227/43,025 (0.5)	128/35,187 (0.4)	42/5451 (0.8)	57/2387 (2.4)	
CHF in 30 days before surgery	62/43,025 (0.1)	37/35,187 (0.1)	10/5451 (0.2)	15/2387 (0.6)	<0.001
History of severe COPD	1071/43,025 (2.5)	744/35,187 (2.1)	183/5451 (3.4)	144/2387 (6.0)	<0.001
Ascites	7/43,025 (0.0)	5/35,187 (0.0)	2/5451 (0.0)	0/2387 (0.0)	0.48
Acute renal failure	32/43,025 (0.1)	21/35,187 (0.1)	4/5451 (0.1)	7/2387 (0.3)	0.003
Bleeding disorders	525/43,025 (1.2)	365/35,187 (1.0)	94/5451 (1.7)	66/2387 (2.8)	<0.001
>10% loss body weight in last 6 months	205/43,025 (0.5)	141/35,187 (0.4)	27/5451 (0.5)	37/2387 (1.6)	<0.001
Disseminated cancer	429/43,025 (1.0)	286/35,187 (0.8)	77/5451 (1.4)	66/2387 (2.8)	0.54
Open wound/wound infection	120/43,025 (0.3)	94/35,187 (0.3)	17/5451 (0.3)	9/2387 (0.4)	<0.001
Mean (SD):					
Total operation time, min	214.42 (93.11)	211.63 (90.01)	215.68 (96.81)	252.76 (117.64)	<0.001
Work relative value unit	31.68 (4.26)	31.53 (3.85)	31.77 (4.90)	33.63 (7.05)	<0.001

CHF, Congestive heart failure; COPD, chronic obstructive pulmonary disease; PRBCs, packed red blood cells.

### Mortality

Overall, the 30-day postoperative mortality rate was 0.2% across the three different age groups. The univariate analysis showed a significant increase in the crude 30-day mortality amongst the groups. It was 0.1% in the <70 years age group, 0.4% for those aged 70–74 years, and 1.3% for those aged >74 years (Table 2; Figure 1).

**Table 2.** Univariate analysis.

Variable		Age group, years				Unadjusted OR (95% CI)			
	All (*N* = 43,025), *n* (%)	<70 (*n* = 35,187), *n* (%)	70–74 (*n* = 5451), *n* (%)	>74 (*n* = 2387), *n* (%)	*P*	70–74 years (*n* = 5451)	*P*	>74 years (*n* = 2387)	*P*
Mortality	106 (0.2)	50 (0.1)	24 (0.4)	32 (1.3)	<0.001	3.11(1.91–5.06)	<0.001	9.55(6.11–14.91)	<0.001
Composite morbidity^a^	2113 (4.9)	1420 (4.0)	324 (5.9)	369 (15.5)	<0.001	1.5(1.24–1.87)	<0.001	4.35(3.84–4.92)	<0.001
Wound	720 (1.7)	495 (1.4)	116 (2.1)	109 (4.6)	<0.001	1.52(1.24–1.87)	<0.001	3.35(5.91–11.70)	<0.001
Cardiac	180 (0.4)	94 (0.3)	34 (0.6)	52 (2.2)	<0.001	2.34(1.58–3.47)	<0.001	8.31(5.91–11.70)	<0.001
Respiratory	387 (0.9)	213 (0.6)	78 (1.4)	96 (4.0)	<0.001	2.38(1.84–3.09)	<0.001	6.88(5.39–8.79)	<0.001
Urinary	289 (0.7)	185 (0.5)	55 (1.0)	49 (2.0)	<0.001	1.93(1.42–2.61)	<0.001	3.96(2.89–5.45)	<0.001
CNS^b^	73 (0.3)	46 (0.2)	16 (0.5)	11 (0.8)	<0.001	2.35(1.33–4.16)	0.003	3.67(1.90–7.10)	<0.001
Thromboembolism	613 (1.4)	444 (1.3)	78 (1.4)	91 (3.8)	<0.001	1.14(0.89–1.45)	0.30	3.10(2.47–3.90)	<0.001
Sepsis	719 (1.7)	459 (1.3)	113 (2.1)	147 (6.2)	<0.001	1.60(1.30–1.97)	<0.001	4.96(4.10–6.01)	<0.001
Bleeding	2875 (6.7)	1860 (5.3)	449 (8.2)	566 (23.7)	<0.001	1.61(1.44–1.79)	<0.001	5.57(5.01–6.19)	<0.001
Return to operating room	701 (1.6)	506 (1.4)	101 (1.8)	94 (3.9)	<0.001	1.29(1.04–1.60)	0.02	2.81(2.25–3.52)	<0.001

^a^Composite morbidity considered positive if any of wound, cardiac, respiratory, urinary, CNS, sepsis or thromboembolism is positive.

^b^Sample size: 25,228.

The multivariate analysis accounted for patient comorbidities such as hypertension, BMI, diabetes, chronic obstructive pulmonary disease, and congestive heart failure. Similarly, the 30-day postoperative mortality risk after RP was associated with older age. In comparison to the <70 years age group, the 70–74 years age group was associated with a 3.05-fold mortality rate increase, whereas the >74 years age group was associated with an 8.52-fold mortality rate increase (Table 3).

**Table 3.** Multivariate analysis.

	Adjusted OR (95% CI)			
Variable	70–74 years (*n* = 5451)	*P*	>74 years (*n* = 2387)	*P*
Mortality	3.05 (1.86–4.99)	<0.001	8.52 (5.36–13.56)	<0.001
Composite morbidity^a^				
Wound	1.49 (1.21–1.83)	<0.001	3.25 (2.63–4.02)	<0.001
Cardiac	2.18 (1.46–3.24)	<0.001	7.45 (5.24–10.60)	<0.001
Respiratory	2.26 (1.74–2.94)	<0.001	5.91 (4.60–7.59)	<0.001
CNS^b^	2.28 (1.28–4.05)	0.005	3.44 (1.76–6.73)	<0.001
Thromboembolism	1.12 (0.88–1.43)	0.35	2.63 (2.08–3.32)	<0.001
Sepsis	1.54 (1.25–1.89)	<0.001	4.64 (3.83–5.63)	<0.001
Bleeding	1.57 (1.41–1.75)	<0.001	5.25 (4.71–5.84)	<0.001

^a^Composite morbidity considered positive if any of wound, cardiac, respiratory, urinary, CNS, sepsis or thromboembolism is positive.

^b^Sample size: 25,228.

### Morbidity

Overall, 2113 patients had at least one perioperative complication. In all, 4% of the <70 years age group had at least one complication in comparison to a 5.9% and 15.5% complication rate in the 70–74 years and the >74 years age groups, respectively.

In the univariate analysis, increased age was associated with an increased risk of cardiac complications (<70 years: 0.3%; 70–74 years: 0.6%; >74 years: 2.2%), wound complications (<70 years: 1.4%; 70–74 years: 2.1%; >74 years: 4.6%), thromboembolism (<70 years: 1.3%; 70–74 years: 1.4%; >74 years: 3.8%), and sepsis (<70 years: 1.3%; 70–74 years: 2.1%; >74 years: 6.2%) (Table 2).

In the multivariate analysis, again confounding variables including patient comorbidities were accounted for, similarly the above results were confirmed. An increased association of age with cardiac morbidities was noted. In comparison to the <70 years age group, the 70–74 years age group was associated with a 2.18-fold cardiac morbidity increase, whereas the >74 years age group was associated with a 7.45-fold cardiac morbidity increase (Table 3).

Similarly, postoperative wound complications increased by 1.49-times and 3.25-times in the 70–74 and >74 years age groups.

When compared with the <70 years age group, the increase in thromboembolic events was statistically insignificant in the 70–74 years age group. Nevertheless, a significant increase was noted in the >74 years age group with a 2.63-fold increase in thromboembolic events when compared to the <70 years age group.

Finally, the risk of postoperative sepsis increased by 1.54-times and 4.64-times in the 70–74 and >74 years age groups, respectively (Figure 1).

## Discussion

Most (64%) of the newly diagnosed PCa cases in the USA were diagnosed in men aged >65 years, while 23% of cases were diagnosed in men >75 years. With the continuous improvement in overall life expectancy, a 70-year-old man’s average life-expectancy is now reaching 13 years [13]. As such, management of PCa should be tailored in a way that ensures optimal management of disease whilst delaying mortality and minimising treatment-related morbidities.

In the USA, 16% of men diagnosed with PCa die despite all available treatments, while PCa is a direct cause of 3% of all male mortality [14]. Furthermore, the disease has an indolent course with a median time of 8 years from biochemical failure to metastasis and 5 years from metastasis to death [15]. For many years now, RP has been the modality of choice for localised PCa in surgically fit men. The aim of surgery is achieving the trifecta of survival, continence preservation, and erectile function preservation [16].

Based on the recommendation of the SIOG, older patients with PCa should be managed according to their general health status and comorbidities rather than their age [4–8]. Several studies revealed an association between an older age and aggressive disease characteristics [9]. In a recent analysis of treatment modalities used in locally advanced PCa, using the Surveillance, Epidemiology, and End Results (SEER) database (2004–2013), revealed that 19.5% of men aged >70 years, underwent a RP as a primary modality for their local disease [17].

In a retrospective analysis of 1110 patients with PCa who underwent RP, Alibhai et al. [18] reported that age is associated with an increased risk of 30-day postoperative mortality and perioperative complications after accounting for comorbidities. Similarly, an analysis of SEER data (1992–1996) showed an increase in postoperative complications with respect to age group, with complication rates of 28%, 31%, and 35% in men aged 65–69, 70–74 and >74 years, respectively. Furthermore, the 30-day mortality rates for those age groups were 0.4%, 0.5% and 0.9%, respectively [19].

Our present results revealed an increasing morbidity as well as mortality rate with increasing age. All in all, the >74 years age group had a significantly higher complication rate than younger patients with a statistically significant higher chance of composite morbidity including the rates of wound infection, cardiac, pulmonary, central nervous system (CNS), urinary, thromboembolism and sepsis. The risk of bleeding was ~3-times higher in the >74 years age group compared to those aged 70–74 years and 4-times higher compared to the <70 years age group. Such discrepancies reiterate the need for careful patient selection based on age upon deciding on treatment modalities for PCa due to the significant differences observed in postoperative morbidity and mortality. Watchful waiting can be an alternative for a selected number of patients. According to the Scandinavian Prostate Cancer Group Study Number 4 (SPCG-4) study, there was no difference between surgical intervention and observation in patients diagnosed with PCa prior to the PSA screening era; patients aged >65 years did not benefit from surgery in terms of years to metastatic disease or cancer-specific mortality over a 15-year follow up [20]. Moreover, the Prostate Cancer Intervention Versus Observation Trial (PIVOT) revealed that surgeries done based on screening PSA for PCa did not significantly reduce cancer-specific or overall mortality rates. Over a 20-year follow-up period, it concluded that compared to observation, RPs caused a higher risk of adverse events yet a lower frequency of treatment for disease progression [21].

AS, based on delaying intervention for select patients until alarming signs of disease progression ensues, is an attractive alternative for treating PCa. The core of this modality is based on the slowly progressing nature of the disease. It also aims at minimising overtreatment of PCa. As shown in the two aforementioned studies, surgery did not significantly alter survival outcomes compared to observation [20,21]. As such, AS decreases the risk of overtreatment through careful patient selection. Criteria for AS include patients with low-risk disease (low-volume Gleason 6 and not more than T2 disease clinically), high level of compliance, and a tolerable anxiety regarding the disease taking into account patient comorbidities and life expectancy [22,23]. It is imperative to mention that AS is discouraged for intermediate- and high-risk PCa.

The limitations of our present study are mostly related to our data. The data are crude and the possibility of subgroup analysis was limited. Cause–effect relationships pertaining to patient characteristics or disease pathology (Gleason grade, PSA levels, stage) were not assessed except those related to age. In addition, subgroup analysis comparing the impact of surgical technique in RP was not done (i.e. open approach vs laparoscopic or robot-assisted). Another caveat of our present study was the inability to assess postoperative continence or potency.

## Conclusion

Our present study sheds light on the effect of age as an independent factor in determining risk of perioperative mortality and morbidity following RP. In conclusion, age by itself should play a significant role in selecting surgical candidates with PCa, whereby older individuals are at higher risk for morbidity and even mortality; hence alternative therapies could be considered.
